# Chikusetsu saponin IVa protects pancreatic β cell against intermittent high glucose-induced injury by activating Wnt/β-catenin/TCF7L2 pathway

**DOI:** 10.18632/aging.102702

**Published:** 2020-01-22

**Authors:** Jia Cui, Jialin Duan, Jianjie Chu, Chao Guo, Miaomiao Xi, Yi Li, Yan Weng, Guo Wei, Ying Yin, Aidong Wen, Boling Qiao

**Affiliations:** 1 Department of Pharmacy, Xijing Hospital, Fourth Military Medical University, Xi’an 710032, Shaanxi, China; 2 Department of Chinese Medicine, School of Life Science, Northwestern University, Xi’an 710032, Shaanxi, China; 3 Department of Pharmacy, Chongqing Dazu District Hospital of Traditional Chinese Medicine, Chongqing 402360, China

**Keywords:** Chikusetsu saponin IVa, Wnt, TCF7L2, β-catenin, glucose fluctuation

## Abstract

Islet β cell mass reduction induced by glucose fluctuation is crucial for the development and progression of T2DM. Chikusetsu saponin IVa (CHS) had protective effects against DM and related injuries. Here we aimed to investigate the role of CHS in β cell injuries and its possible mechanism involved. Isolated rat islets, βTC3 cells and T2DM mice were used in this study. The results showed that CHS restored the secretion activity, promoted β cell survival by increasing β cell proliferation and decreasing apoptosis which induced by intermittent high glucose (IHG). *In vivo*, CHS protected β cell apoptosis to normalize blood glucose and improve insulin sensitivity in DM mice. Further studies showed that CHS activated Wnt3a signaling, inhibited HBP1, promoted β-catenin nuclear translocation, enhanced expressions of TCF7L2, GIPR and GLP-1R, inhibited p53, p27 and p21. The protective effect of CHS was remarkably suppressed by siRNAs against TCF7L2 or XAV-939 (a Wnt/β-catenin antagonist) *in vitro* and in β-catenin^-/-^ mice. In conclusion, we identified a novel role of CHS in protecting β cell survival and regeneration by mechanisms involving the activation of Wnt3a/β-catenin/TCF7L2 signaling. Our results indicated the potential value of CHS as a possible intervention drug for T2DM.

## INTRODUCTION

Diabetes mellitus (DM), which is caused by defective insulin secretion or insulin sensitivity, has been a large public health burden. According to statistical data, there are 200 million diabetics globally, and this will be doubled by 2025. [1]. Pancreatic islet β cells have crucial effects in controlling the glucose homeostasis through precision regulating insulin release level [2]. Under physiological conditions, pancreatic β cell mass is dynamic controlled by glucose and other factors. However, this ability of the regulation may be attenuated in DM and lead to sustained high glucose (SHG) and intermittent high glucose (IHG) [3]. The patients with long duration of DM will be prone to show a higher glucose variation [4, 5]. IHG has been widely suggested with more toxic effects on many kinds of cells, and more dangerous for the development of DM and DM-related complications than SHG [6]. These evidences showed that IHG induce β cells apoptosis and affect the function of pancreatic β cells. The transcription factor 7-like 2 (TCF7L2) gene, also known as T-cell factor 4 (TCF4), is a member of TCF/lymphoid-enhancing factor family [7]. Previous study had confirmed that TCF7L2 serves as a nuclear receptor for β-catenin, and activates many Wnt downstream cascades [8]. Wnt signaling pathway is a indispensable part for the development of endocrine pancreas and functional regulation of β cells, including insulin secretion, cell survival and proliferation [9, 10]. In pancreatic islet cells, TCF7L2 influences β cells functions through affecting β cells growth and differentiation [11]. Some case reports had shown that the role of TCF7L2 polymorphisms in inducing T2DM had cross talk relationships between Wnt and other signaling pathways (such as GLP-1 signaling) in the pancreatic β cells [12, 13]. Because of the important role of Wnt/TCF7L2 pathway in the β cells apoptosis, it may play some roles in the IHG induced injuries. However, it remains largely unknown.

**Figure 1:**
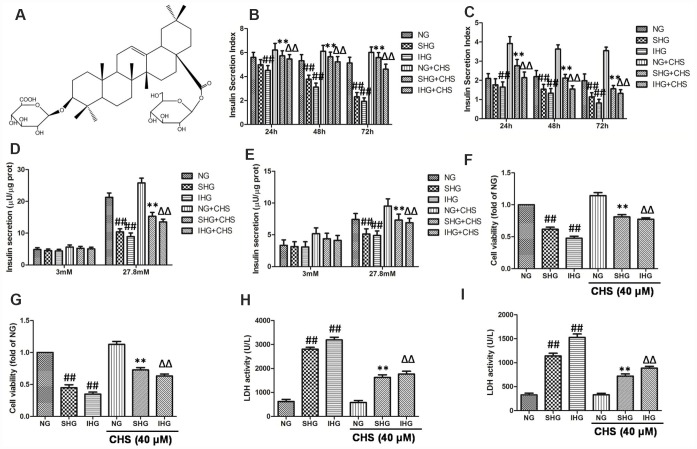
**CHS protected against proliferation and cytotoxicity of islet cells from IHG.** (**A**) The chemical structure of CHS. Molecular weight: 794. Molecular formula: C42H66O14. The glucose stimulated insulin secretion in primary pancreatic islet cells (**B**) and βTC3 cells (**C**) were measured by an insulin RIA kit after incubation for 24, 48 and 72 h. The insulin secretion levels in primary pancreatic islet cells (**D**) and βTC3 cells (**E**) in response to 3.0 mM and 27.8 mM glucose stimulation. Cell viability of primary pancreatic islet cells (**F**) and βTC3 cells (**G**) was measured by a CCK-8 assay. Cytotoxicity in primary pancreatic islet cells (**H**) and βTC3 cells (**I**) was measured by an LDH assay. Data are representative of three independent experiments. ^##^*P*<0.01 vs. NG treatment group, ^**^P<0.01 vs. SHG treatment group, ^ΔΔ^*P*<0.01 vs. IHG treatment group.

As an ancient traditional Chinese medicine, *Aralia taibaiensis* has been used in treating DM and other metabolic diseases for a long time in China, Korea, and Japan [14]. In previous studies, we found that triterpenoid saponins in Aralia taibaiensiis exhibited protective effects against high glucose induced islet cells and myocardial cell apoptosis, and protected the islet injuries in DM rats [15, 16]. However, its mechanism is largely unknown for us. Chikusetsu saponin IVa (Figure 1A, CHS) was a triterpenoid saponin isolated from *Aralia taibaiensis* and showed beneficial effects in DM and related injuries [14, 17]. These results suggested that CHS is a potential drug for DM. However, whether CHS was effective in IHG inducing islet injuries was still unknown. Therefore, this study was designed to determine whether CHS could protect IHG inducing islet injuries and elucidate the hypothesis that Wnt/TCF7L2 might be involved in the protection of CHS.

## RESULTS

### CHS protected islet cells from IHG induced functional and variability impairment

The glucose stimulated insulin secretion (GSIS) in the primary pancreatic islet cells and βTC3 cells were measured by an insulin RIA kit after incubation for 24, 48 and 72 h, and the insulin secretion indexes were calculated as the GSIS results. As the results showed in primary pancreatic islet cells (Figure 1B) and βTC3 cells (Figure 1C), compared with NG treatment group, SHG treatment resulted in a significant decrease of insulin secretion index in a time-dependent manner, and IHG treatment was more pronounced (P<0.01). After incubation for 72 h, insulin release levels induced by 27.8 mM glucose were significantly decreased in SHG and IHG treatment group, and IHG group showed more evident in primary pancreatic islet cells (Figure 1D) and βTC3 cells (Figure 1E). However, CHS treatment groups had higher insulin secretion index at this three time points compared with IHG or SHG treatment group and 27.8 mM glucose induced insulin release levels were much more than the IHG or SHG treatment group. These results indicated that the function of islet cells were impaired by IHG and SHG treatment however CHS protected IHG induced functional impairment, restored the secretion activity of islet cells.

The effects of IHG treatment on cells variability were measured by the CCK8 kit. As the results showed in primary pancreatic islet cells (Figure 1F) and βTC3 cells (Figure 1G), SHG and IHG induced significantly lower proliferation than the NG group, and IHG group was lower than SHG group. In addition, cell proliferation in the CHS treatment groups were significantly higher than that in the IHG or SHG group (*P*<0.01), suggesting that CHS protected cell proliferation from IHG intervention.

**Figure 2:**
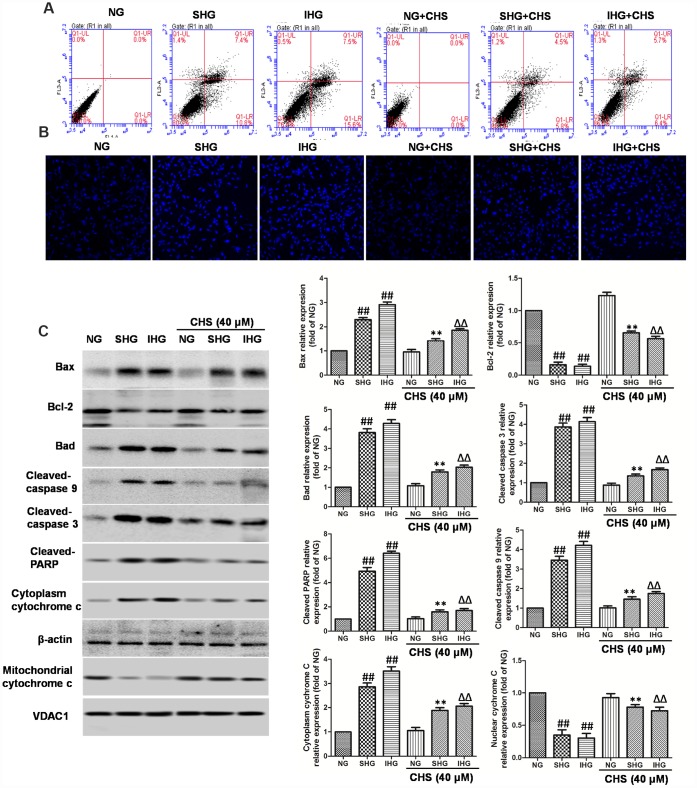
**CHS protected against apoptosis of islet cells from IHG.** (**A**) The apoptosis rate of βTC3 cells was assessed using flow cytometry with Annexin-V/PI staining. (**B**) The cell nuclear morphology of βTC3 cells was measured by DAPI staining. Fluorescence microscope (200×) images. (**C**) The apoptosis related proteins expression levels were measured by western blotting according to the methods 2.12. Data are representative of three independent experiments. ^##^*P*<0.01 vs. NG treatment group, ^**^P<0.01 vs. SHG treatment group, ^ΔΔ^*P*<0.01 vs. IHG treatment group.

To further investigate the protective effect of CHS, the LDH levels were measured in primary pancreatic islet cells (Figure 2H) and βTC3 cells (Figure 2I). The levels of LDH leakage in the IHG and SHG groups were significantly increased and were higher than those in the NG group (*P*<0.01). However, the levels of LDH leakage in the IHG+CHS and SHG+CHS groups had lower LDH leakage (*P*<0.01). There was no significant difference between IHG+CHS and SHG+CHS group. These results indicated that IHG or SHG caused cytotoxicity in primary pancreatic islet cells and βTC3 cells, and CHS treatment reduced LDH leakage significantly.

### CHS protected cells from IHG induced apoptosis

To determine whether CHS protected cells from IHG or SHG induced apoptosis, Annexin-V and PI double staining was used. As the results showed in Figure 2A, IHG and SHG induced higher apoptosis rate than that in the NG group. In the CHS treatment group, the apoptosis rate was decreased significantly compared with IHG and SHG treatment group (Figure 2A). Next, the nuclear morphology was stained by DAPI to assess the nuclear shape and chromatin integrity changes. As the results showed in Figure 2B, the nuclei had a normal shape and uniformly stained chromatin, after incubation with SHG and IHG for 72h, cells revealed the marked condensed and fragmented chromatin. In CHS pretreatment group, the number of abnormal nuclear staining was much lower than that in SHG or IHG group, suggesting CHS treatment could protect cell from apoptosis (*P*<0.01).

Next, we studied the possible caspase-dependent pathway through testing the expression of Bax, Bad, Bcl-2, cleaved-caspase-9, cleaved-caspase-3 and poly (ADP-ribose) polymerase (PARP) enzyme (Figure 2C). SHG or IHG treatment enhanced the expression of Bax, Bad, cleaved-caspase-9, cleaved-caspase-3 and cleaved-PARP-1, and inhibited the expression of Bcl-2. In IHG+CHS and SHG+CHS group, the expression of Bax, Bad, cleaved-caspase-9, cleaved-caspase-3 and cleaved-PARP were significantly inhibited and Bcl-2 was increased (*P*<0.01). Cytochrome c is a member of the caspase apoptosis pathway, and is one of the rate-limiting stages in apoptotic cell death. In this study, we tested the levels of cytochrome c in mitochondrial and cytosolic fractions. Western blot results showed that cytochrome c release from mitochondria was increased significantly in SHG or IHG treatment group compared with the NG group, however, the levels of cytochrome c in cytosolic fraction of CHS treatment group was significantly decreased compared with SHG or IHG group (P<0.01). These results suggested that SHG or IHG induced apoptosis through caspase-9 and -3 intrinsic apoptotic pathways, and CHS inhibited these effects.

### CHS protected cells from IHG induced cell cycle arrest

**Figure 3:**
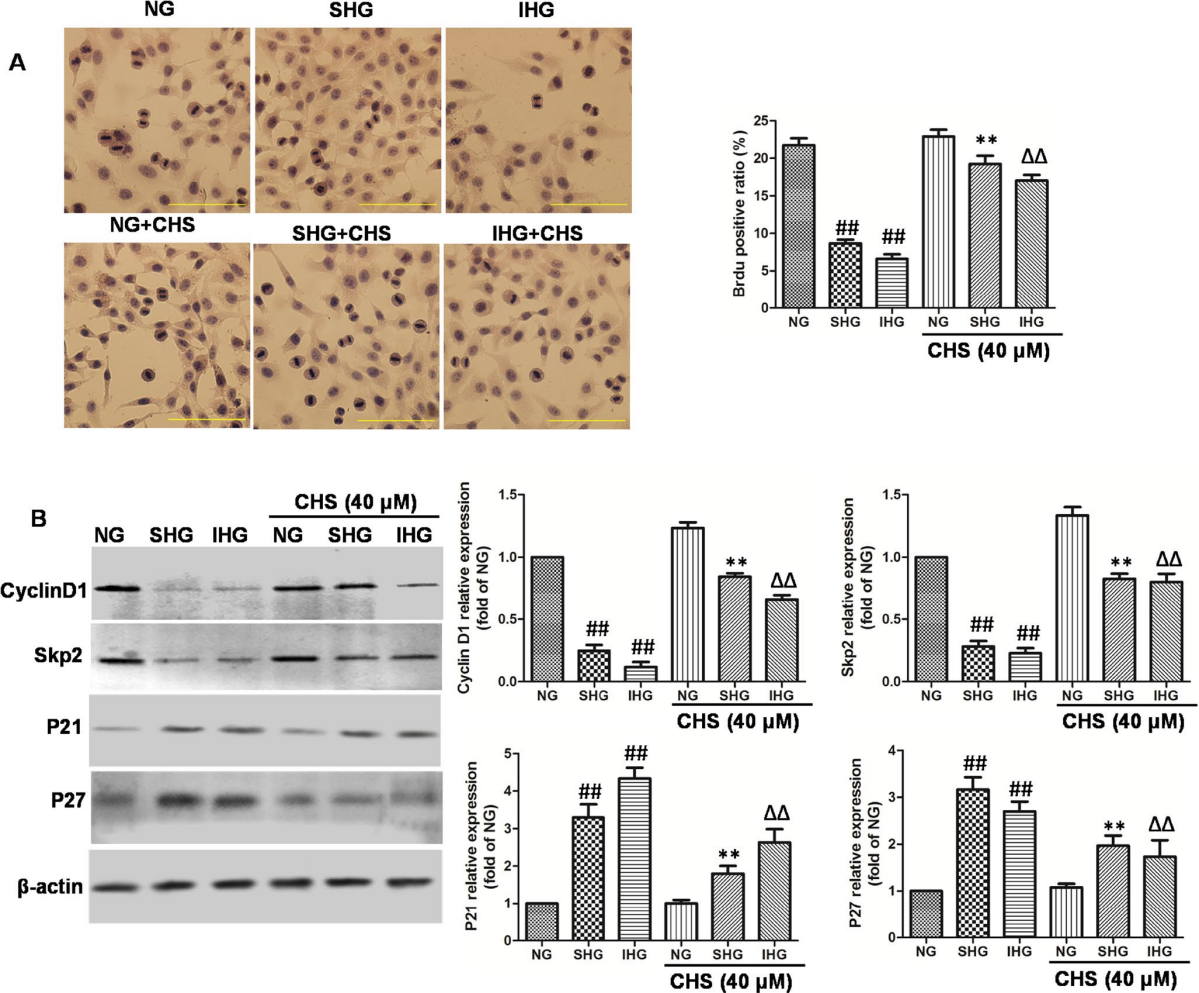
**CHS protected against cell cycle arrest of islet cells from IHG.** (**A**) The βTC3 cell proliferation was measured by a BrdU labeling and detection kit. Inverted microscope (200×) images. (**B**) The cell cycle related proteins (Cyclin D1, Skp2, p21, andp27) were measured by western bolting. Data are representative of three independent experiments. ^##^*P*<0.01 vs. NG treatment group, ^**^P<0.01 vs. SHG treatment group, ^ΔΔ^*P*<0.01 vs. IHG treatment group.

BrdU is a thymidine analog that incorporates DNA of dividing cells during the S-phase of the cell cycle. So, BrdU is always used for monitoring cell proliferation. In this study, BrdU immunohistochemistry was performed in βTC3 cells. Results of immunohistochemical analysis showed that the ratio of Brdu positive β-cells in SHG and IHG group were significantly lower than that in NG group (Figure 3A). However, the positive ratio of Brdu in CHS treatment group was significantly higher than that in SHG or IHG groups (*P*<0.01).

To determine the possible mechanism through which CHS promoted the proliferation of βTC3 cells, the protein expression levels of cyclinD1, skp2, p21, and p27 were analyzed by Western blot (Figure 3B). The expression of cyclinD1 and skp2 were significantly downregulated in the IHG and SHG treatment group (*P*<0.01), Meanwhile the expression levels of p21 and p27 were markedly increased by IHG and SHG (*P*<0.01). Whereas we found that CHS induced the expression of cyclinD1 and skp2, and inhibited the expression of p21 and p27 obviously. These results indicated that CHS might promote the proliferation activity of βTC3 cells through regulating the cyclin related proteins.

### CHS induced the TCF7L2 expression in islet cells

**Figure 4:**
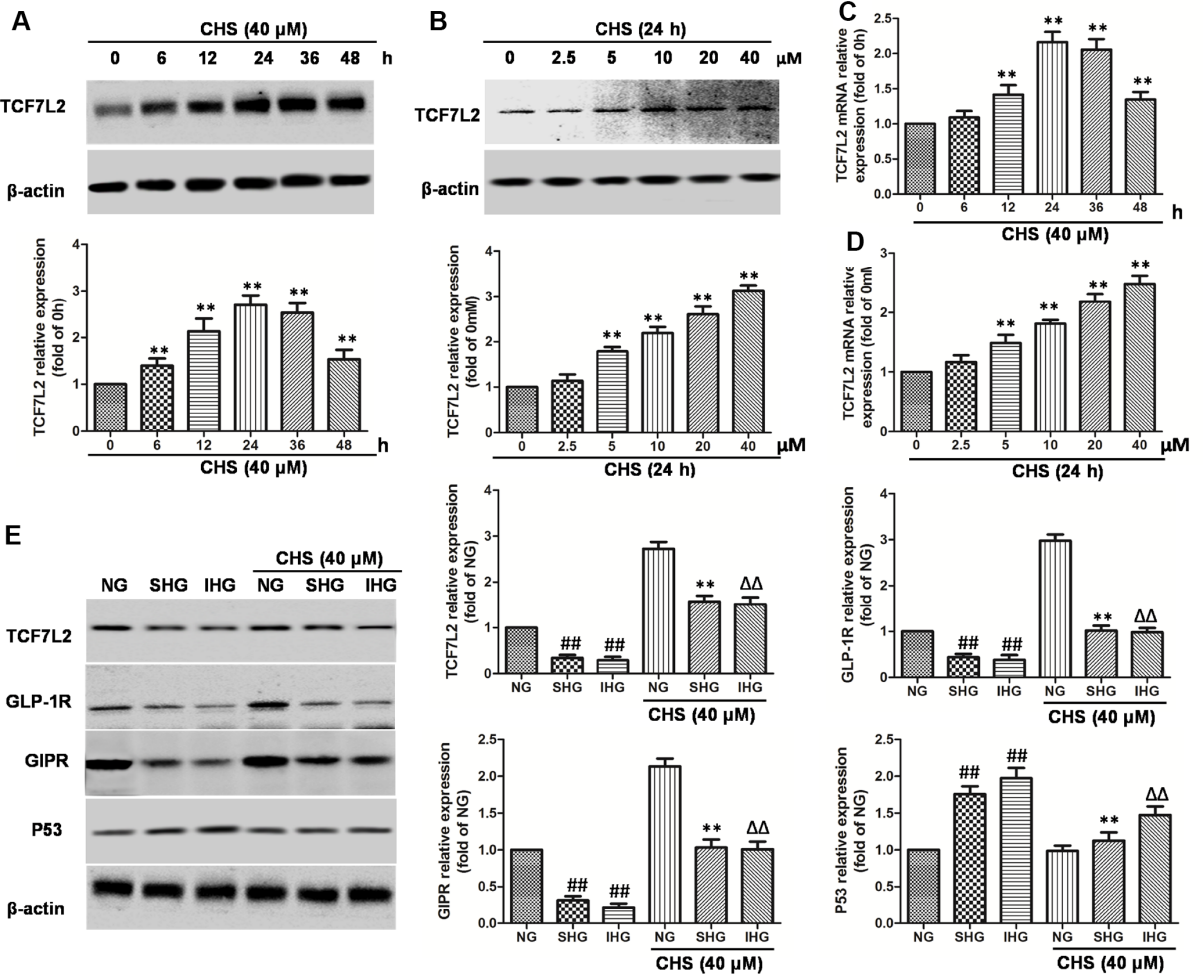
**Effects of CHS on the TCF7L2 expression levels.** (**A**) βTC3 cell was incubated with CHS (40μM) for series of times (0, 6, 12, 24, 36, 48h), then the TCF7L2 protein expression levels was measured by western blotting. (**B**) βTC3 cell was incubated with CHS (0, 2.5, 5, 10, 20, 40μM) for 24h, then the TCF7L2 protein expression levels was measured by western blotting. βTC3 cell was incubated with different doses of CHS (**C**) or different times (**D**), the mRNA levels of TCF7L2 was measured by RT-PCR. (**E**) Effects of CHS on the protein expression levels of TCF7L2, GLP-1R, GIPR and P53 after IHG or SHG treatment. Data are representative of three independent experiments. ^##^*P*<0.01 vs. NG treatment group, ^**^P<0.01 vs. SHG treatment group, ^ΔΔ^*P*<0.01 vs. IHG treatment group.

TCF7L2 mRNA levels are abnormal expressed in islet cells correlated with T2DM. To confirm whether the protective effect of CHS was through upregulating TCF7L2, the mRNA and protein expression levels of TCF7L2 were analyzed. As the results showed in Figure 4A–4D, the mRNA and protein expression levels of TCF7L2 were increased in a dose and time-dependent manner by CHS treatment. The results also showed that SHG or IHG decreased protein expression of TCF7L2, as well as the downstream proteins, GLP-1R and GIPR (Figure 4E) and increased the expression of p53. CHS treatment significantly restored the impaired TCF7L2 expression in SHG or IHG treated islets, and increased the expression of GLP-1R and GIPR, decreased p53 expression.

**Figure 5:**
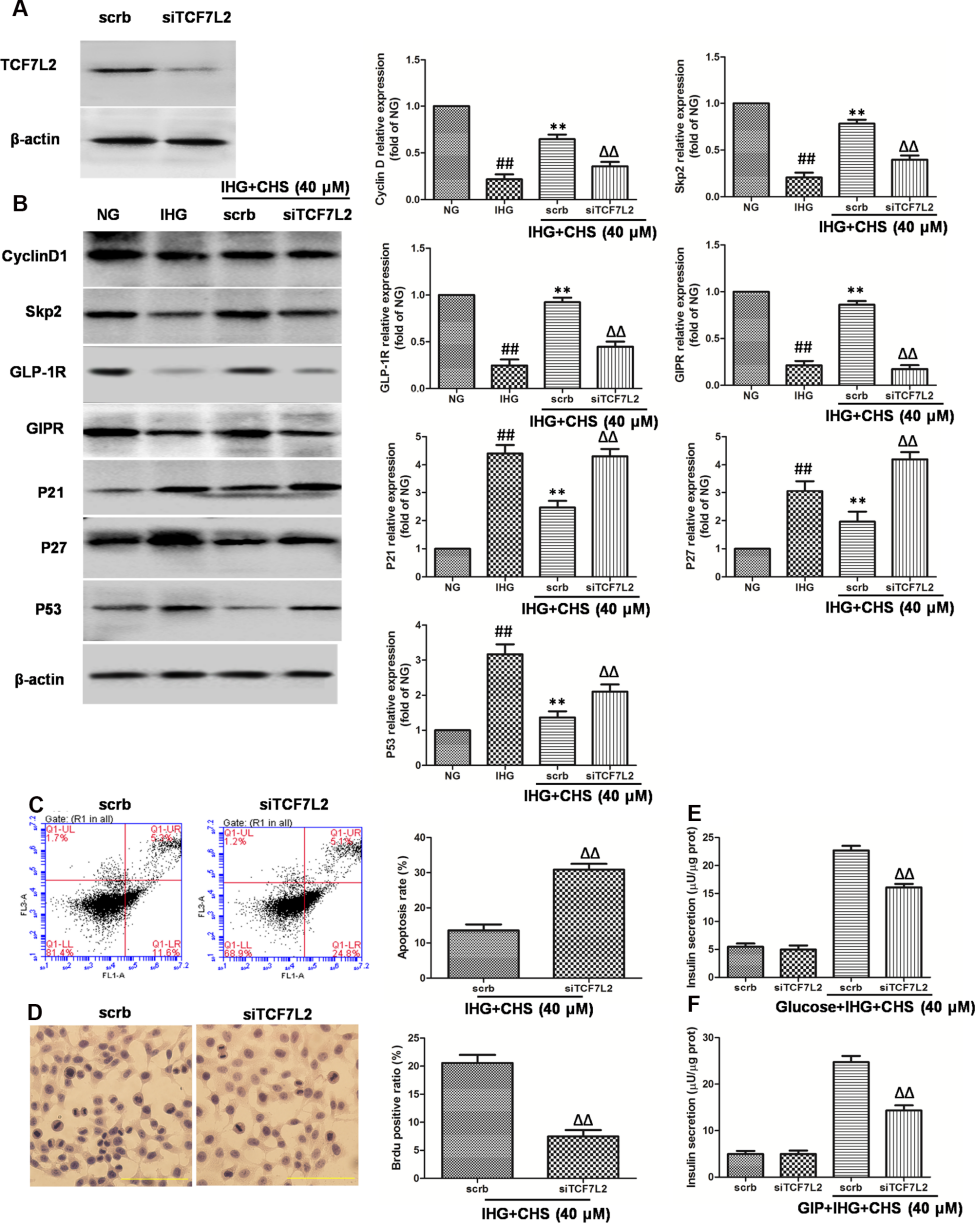
**Effects of CHS might through TCF7L2 pathway.** (**A**) The protein expression of TCF7L2 after siRNA transfection target TCF7L2. (**B**) The protein expression levels of TCF7L2 downstream proteins after inhibition of TCF7L2 by TCF7L2 siRNA. (**C**) The apoptosis rate of βTC3 cell was measured by flow cytometry after inhibition of TCF7L2 by TCF7L2 siRNA. (**D**) The BrdU immunohistochemical staining was performed after inhibition of TCF7L2 by TCF7L2 siRNA. (**E**) The effects of siTCF7L2 on the glucose induced insulin secretion. (**F**) The effects of siTCF7L2 on the GLP-1 induced insulin secretion. Data are representative of three independent experiments. ^##^*P*<0.01 vs. NG treatment group, ^**^P<0.01 vs. IHG treatment group, ^ΔΔ^*P*<0.01 vs. scrb treatment group.

To further investigate the role of TCF7L2 in CHS’s effects, we used siRNA to knock down the TCF7L2 gene. SiTCF7L2 caused a significant 77% reduction of TCF7L2 expression compared with the scrambled control siRNA (scrb) (Figure 5A). SiTCF7L2 resulted in the downregulation of GLP-1R and GIPR which increased by CHS treatment, and also caused increasing of p53 and p21 expression (Figure 5B). Compared with CHS+IHG+scrb group, the expression of cyclinD1 and skp2 were decreased and the expression of p21 and p27 were increased in CHS+IHG+siTCF7L2 group. The anti-apoptosis and proliferation promotion effect of CHS were also abolished by siTCF7L2 (Figure 5D and 5E). We also found that siTCF7L2 caused a 1.4 fold reduction in glucose induced insulin secretion and a 1.8 fold reduction in GLP-1 induced insulin secretion compared with CHS+IHG+ scrb group (Figure 5F and 5G).

### The beneficial effects of CHS were partially dependent on the Wnt/TCF7L2 signaling pathway

**Figure 6:**
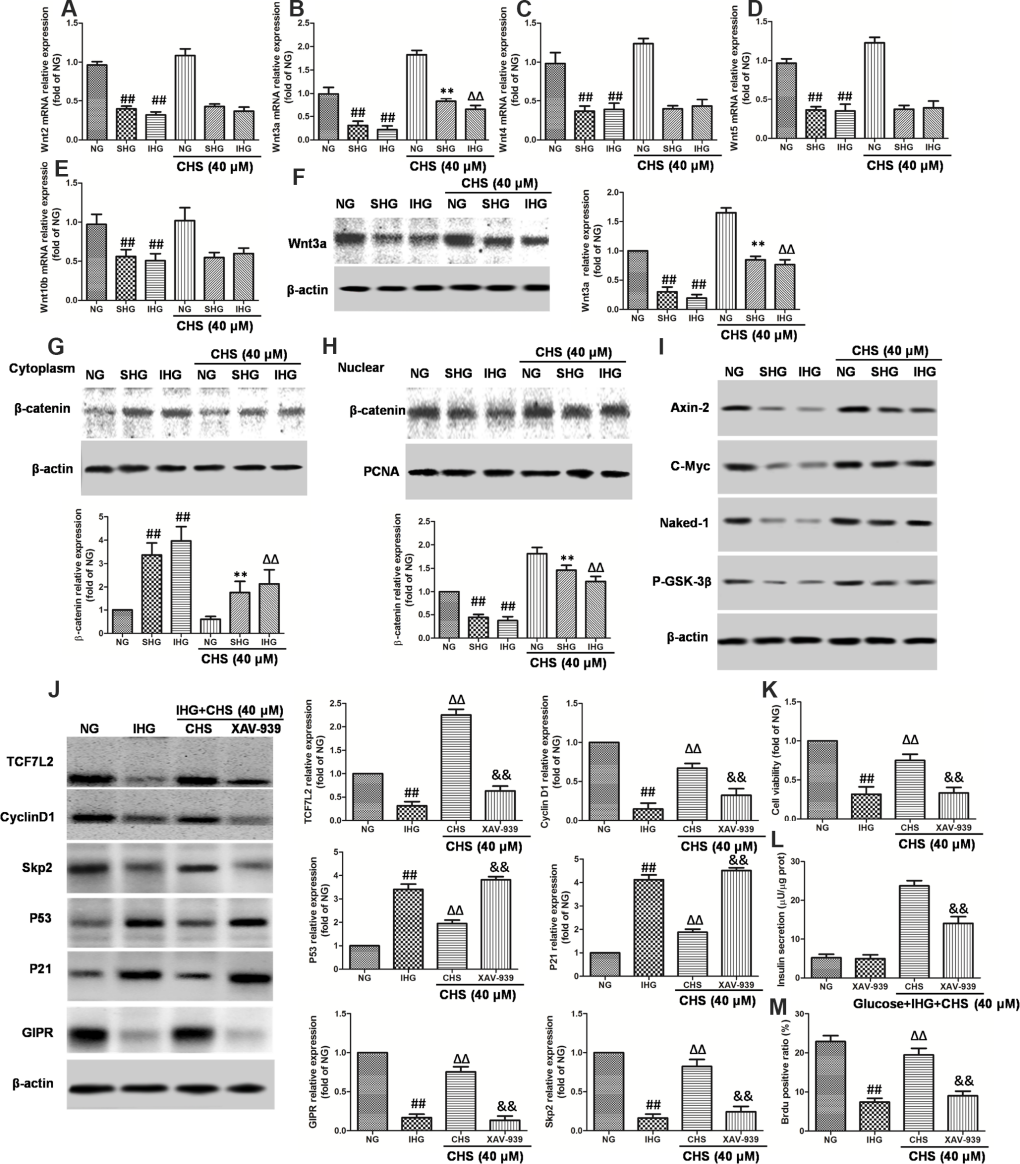
**Involvement of Wnt/TCF7L2 signaling in the protective effect of CHS on islets β cell. βTC3 cells were treated with CHS and IHG or SHG, then the RNA was extracted by TRIZOL.** The mRNA expression levels of Wnt2 (**A**), Wnt3a (**B**), Wnt4 (**C**), Wnt5a (**D**), and Wnt10b (**E**) were measured by RT-PCR. (**F**) The protein expression levels of Wnt3a was measured by western blotting after different treatments. The effects of CHS on β-catenin expression levels in the cytoplasm (**G**) and nuclear (**H**) were measured by western blotting. (**I**) The effects of CHS on protein expression levels of Axin-2, c-Myc, Naked-1 and P-GSK-3β. βTC3 cells were treated with CHS and XAV-939 (a Wnt/β-catenin antagonist, 10μM), and then subjected to IHG. (**J**) The protein expression levels of TCF7L2, cyclin D1, skp2, p53, p21 and GIPR were measured by western blotting. (**K**) Cell variability was measured by CCK8 assay. (**L**) Insulin secretion levels was measured by an insulin RIA kit. (**M**) BrdU positive ratio was calculated from the BrdU positive cell numbers vs total cell numbers. Data are representative of three independent experiments. ^##^*P*<0.01 vs. NG treatment group, ^**^P<0.01 vs. IHG treatment group, ^ΔΔ^*P*<0.01 vs. scrb treatment group, ^&&^*P*<0.01 vs.CHS treatment group.

Wnt proteins are a group of soluble factors that control many cell processes, such as self-renewal, apoptosis, and cell cycle. To explore the possible mechanism for the protective effects of CHS in IHG induced islet cells injury, Wnt mRNA expressions were analyzed. We observed that IHG or SHG caused significant decrease in the expression of Wnt2, Wnt3a, Wnt4, Wnt5a, and Wnt10b compared to that of NG treatment group. CHS treatment significantly increased the expression of Wnt3a among all of the Wnts, raising the possibility that the Wnt proteins were involved in the CHS treatment (Figure 6A–6E). The western blot results also showed that the expression of Wnt3a was increased by CHS treatment (Figure 6F).

When the Wnt proteins are secreted, they bind to its receptors, and induce the expression of its downstream. Thus, the nuclear translocation of β-catenin was analyzed. As the results showed in Figure 6G and 6H, the western blot results showed that SHG and IHG treatment induced the β-catenin predominantly accumulated in the cytoplasmic fraction. However, CHS induced a significant increase of β-catenin levels in the βTC3 cells nuclear whereas the protein levels of β-catenin in cytoplasm was deceased (*P*<0.05). We also analyzed the protein expression of Axin-2, c-Myc, Naked-1 and the phosphorylation levels of GSK-3β and found that the expression of Axin-2, c-Myc, Naked-1 were decreased in IHG and SHG group, and also the P-GSK-3β levels (Figure 6I). However, CHS increased the expression of Axin-2, c-Myc, Naked-1 and P-GSK-3β levels significantly (*P*<0.05).

The next question was how CHS upregulated TCF7L2 expression. It had been reported that Wnt/β-catenin plays an important role in TCF7L2 transcriptional activation. The regulation effects of CHS on Wnt related proteins had been found before, next, a Wnt/β-catenin antagonist XAV-939 was used to block the β-catenin-associated effects. As the results showed, XAV-939 had little effect on βTC3 cells, but it had synergistic effect with IHG effects. After blocking β-catenin, the expression of TCF7L2, cyclinD1 and Skp2 were inhibited, p21 and p53 were increased (Figure 6J), the cell viability and glucose induced insulin secretion levels were decreased significantly compared with CHS treatment group (Figure 6K and 6L). We also found that the ratio of Brdu positive β cells in XAV-939 treatment group was significantly lower than that in CHS treatment group (Figure 6M). These results suggested that the beneficial effects of CHS might through the Wnt/TCF7L2 signaling pathway.

### CHS regulated the TCF7L2 signaling pathway through inhibiting HBP1

**Figure 7:**
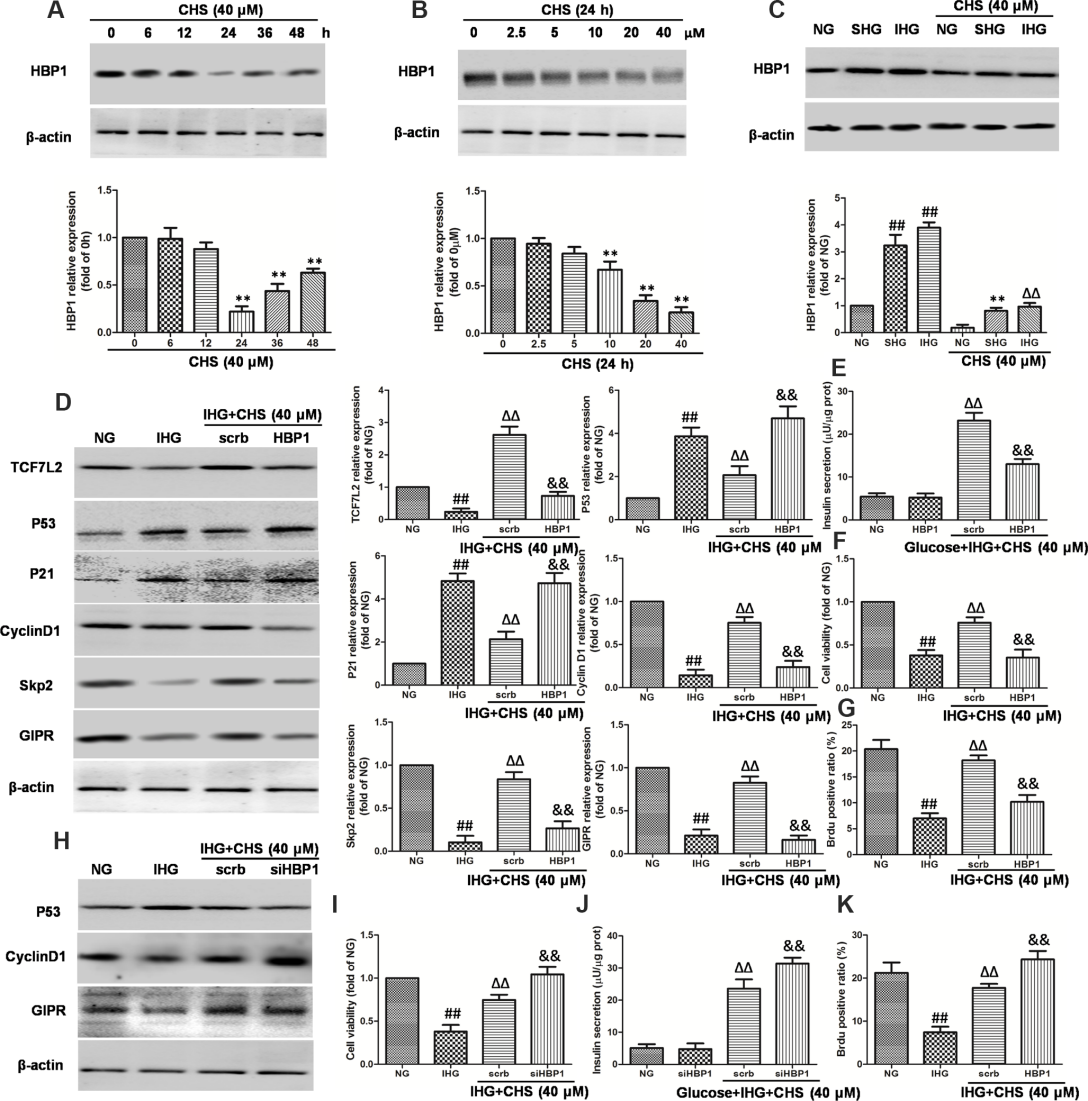
**Effects of CHS on HBP1 expression in islets β cell.** (**A**) Time dependent effects of CHS on the expression HBP1. (**B**) Dose dependent effects of CHS on the expression HBP1. ^**^P<0.01 vs. 0h or 0mM treatment group. (**C**) Effects of CHS on HBP1 expression after IHG or SHG treatment. βTC3 cells were transfected with the empty vector pcDNA3 (scrb) or vectors encoding HA-tagged wild-type HBP1 (HBP1) at 30nM, and then cells were exposed to different treatments as indicated. (**D**) The protein expression levels of TCF7L2, GIPR, cyclinD1, Skp2, p53 and p21 were measured by western blotting. (**E**) Insulin secretion levels was measured by an insulin RIA kit. (**F**) Cell variability was measured by CCK8 assay. (**G**) BrdU positive ratio was calculated from the BrdU positive cell numbers vs total cell numbers. βTC3 cells were transfected with scrb and siHBP1 (30nM) for 24h, and then cells were exposed to different treatments as indicated. (**H**) The protein expression levels of GIPR, cyclinD1, and p53 were measured by western blotting. (**I**) Cell variability was measured by CCK8 assay. (**J**) Insulin secretion levels was measured by an insulin RIA kit. (**K**) BrdU positive ratio was calculated from the BrdU positive cell numbers vs total cell numbers. Data are representative of three independent experiments. ^##^*P*<0.01 vs. NG treatment group, ^ΔΔ^*P*<0.01 vs. IHG treatment group, ^&&^*P*<0.01 vs.scrb treatment group.

HBP1 and LEF-1 are two HMG-box proteins and have opposite effects on transcriptional output. In this study, we hypothesized that IHG induced the expression of HBP1 and then repressed LEF/TCF activity, thus inhibited the Wnt pathway. We first analyzed the effects of CHS on expression of HBP1. As the results in Figure 7A and 7B, treatment with CHS caused significantly reduction of HBP1 expression in a dose and time dependent manner (*P*<0.05). In cells treated with SHG and IHG, the expression of HBP1 was found to be increased significantly, whereas CHS induced a significantly decrease of HBP1 expression compared with SHG or IHG group (Figure 7C). To further analyze the role of HBP1 in the regulation of TCF7L2 and CHS’s effects, HBP1 was overexpressed and knockdown by lentiviral infection. Overexpression of HBP1 caused dramatic decrease of TCF7L2, GIPR, cyclinD1 and skp2, increase of p53 and p21 expression (Figure 7D), and thus abolished the effects of CHS on cell viability, glucose induce insulin release and BrdU positive ratio (Figure 7E–7G). Whereas knockdown of HBP1 had the same effects with CHS on the proteins expression (Figure 7H) and cell viability, and had a synergistic effect with CHS (Figure 7I–7K). These results suggested that CHS induced the expression of TCF7L2 through inhibiting HBP1.

### CHS protected β cell injury *in vivo* through HBP1/Wnt/ TCF7L2 pathway

**Figure 8:**
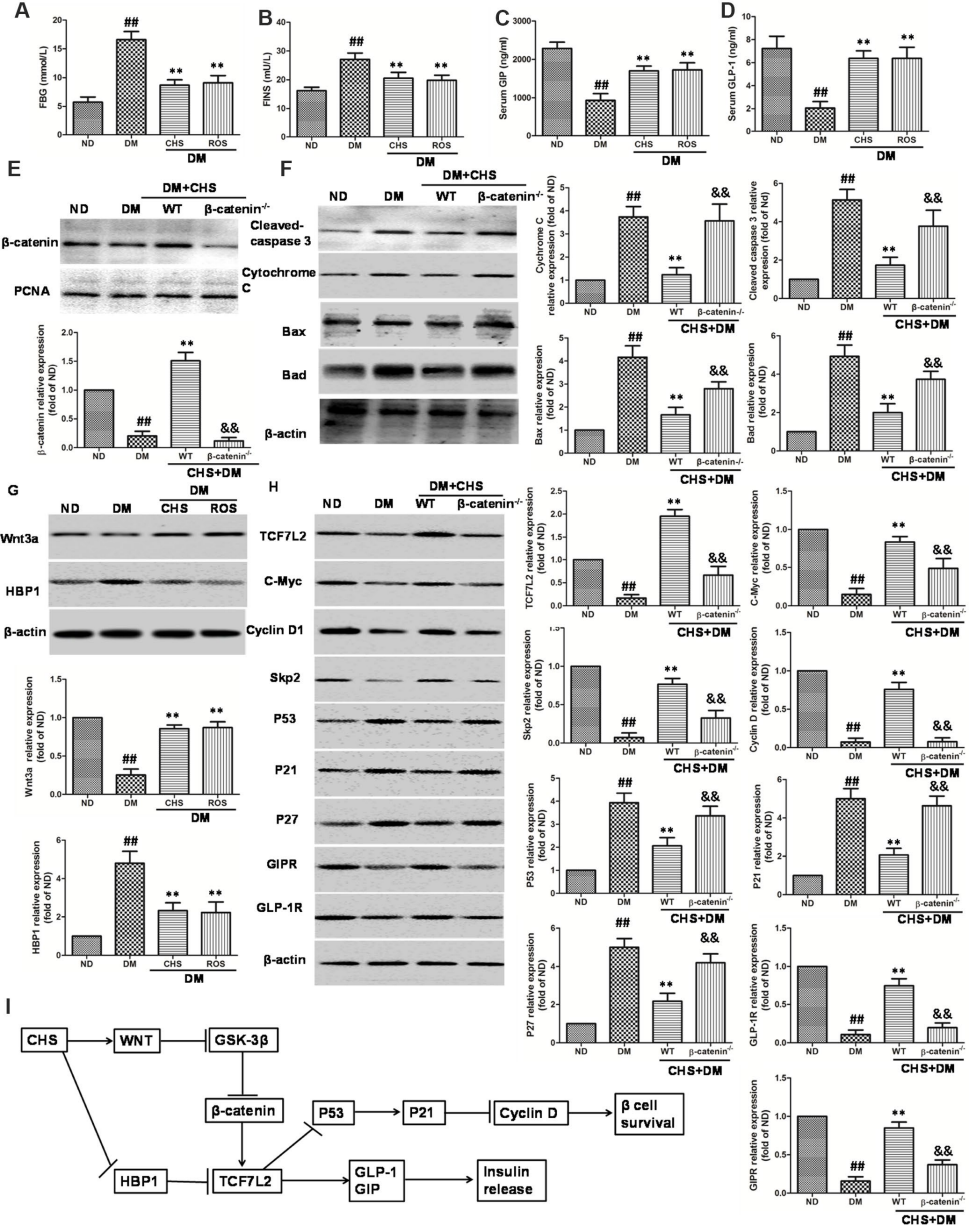
**Effect of CHS on the HBP1/Wnt/ TCF7L2 pathway in vivo.** DM model was induced by HFD and STZ, and the CHS (120 mg/kg) or positive control rosiglitazone (Ros, 2 mg/kg) was given through intragastric administration one time every day for 30days. FBG (**A**), FINS (**B**), GIP (**C**) and GLP-1 (**D**) levels in serum were measured using relative kits. (**E**) Effects of CHS on the expression levels of β-catenin in nuclear. (**F**) Effects of CHS on the expression levels of apoptosis related proteins (cleaved-caspase 3, bax, bad, cytochrome C) in WT and β-catenin^-/-^ DM mice. (**G**) Effects of CHS on the expression levels of Wnt3a and HBP1 in DM mice. (**H**) Effects of CHS on the expression levels of TCF7L2 related proteins (TCF7L2, c-Myc, cyclinD1, Skp2, p53, p21, GLP-1R, GIPR and p27) in WT and β-catenin^-/-^ DM mice. Data are representative of three independent experiments. ^##^*P*<0.01 vs. ND mice group, ^**^P<0.01 vs. DM mice group, ^&&^*P*<0.01 vs. WT DM mice group. (**I**) Potential mechanism underlying the protective effects of CHS on IHG induced cell injuries in DM mice and pancreas islet cells.

To confirm the *in vitro* effects of CHS, a T2DM mouse model was used. The diabetes mice showed a marked increase of FBG and FINS compared with the levels in normal-diet (ND) mice and CHS administration decreased FBG and FBS levels (Figure 8A and 8B). The serum levels of GLP-1 and GIP were also increased after CHS treatment compared with these in T2DM mouse (Figure 8C and 8D). Further, the β-catenin knockdown (β-catenin^-/-^) mice were used to verify the results *in vitro*. As the results showed in Figure 8E, the nuclear β-catenin level was significantly decreased in β-catenin^-/-^ mice. And the apoptosis related proteins including cleaved-caspase 3, bax, bad, cytochrome C were significantly increased in islet tissue, and CHS inhibited these proteins expression to a significant level in WT mice (Figure 8F). Compared with WT mice, cleaved-caspase 3, bax, bad, cytochrome C expression levels were significantly increased in β-catenin^-/-^ mice (*P*<0.01).

As the results showed in Figure 8G, the level of Wnt3a was decreased and HBP1 level was increased in DM mice which compared with ND mice. However, CHS treatment significantly increased the Wnt3a levels and decreased HBP1 level (*P*<0.01). The expression levels of TCF7L2, c-Myc, cyclinD1, skp2, p53, p21 and p27 in islet tissue were also measured by western blotting. The results of western blotting showed significant reduction of TCF7L2, c-Myc, cyclinD1, skp2 and increase of p53, p21 and p27 in the islets of T2DM mouse, while CHS could reverse these changes (Figure 8H). The protein levels of GLP-1R and GIPR were also increased after CHS treatment in WT mice. Compared with WT mice, the expression levels of TCF7L2, c-Myc, cyclinD1, skp2, GLP-1R and GIPR were significantly decreased and p53, p21 and p27 were increased in β-catenin^-/-^ mice (*P*<0.01).

## DISCUSSION

Glucose toxicity induced β cell apoptosis is an important cause of functional β cell loss and contributes to further β cell dysfunction and number reduction, thus leads to the onset and development of diabetes [18]. Generally, the glucose toxicity is characterized as sustained high glucose (SHG) which induced impairment of beta cell functions and survival. In health people with normal pancreas function, the glucose concentrations in serum is strictly controlled and fluctuate in a narrow range, but the plasma glucose concentration often changes markedly in the diabetes during a single day [19, 20]. Additionally, more and more studies have pointed out that intermittent high glucose (IHG) is more dangerous for further β cell dysfunction than SHG [21]. Novel agents designed to protect the β cell injuries induced by IHG are urgently needed.

Natural herbs are important resources for the discovery and development of antidiabetic drug [22]. Previous study had showed that triterpenoid saponins from natural herbs have antidiabetic activity such as astragalosides, ginsenosides, aralia taibaiensis and panax japonicas. Chikusetsu saponin IVa (CHS) was a triterpenoid saponin isolated from *panax japonicas* and *Aralia taibaiensis* and had an excellent ability in promoting insulin release [16]. Our previous studies showed that CHS protected hyperglycemia-induced myocardial injury by activating the SIRT1/ERK1/2/Homer1a pathway [17, 23]. However, the effects of CHS on β cell survival and function were still largely unknown to us. In this study, we aimed to investigate the protective effects of CHS against IHG induced injuries and illuminate the role of HBP1/Wnt/ TCF7L2 in this process.

Previous researches had showed that human pancreatic islets incubated with IHG (5.5 and 16.7 mmol/l) caused significantly reduction of the glucose stimulated insulin secretion index [24]. In rat islets and INS-1 cell experiment, IHG induced a more significant impairment of insulin release response than SHG, and a lower GSIS [25]. In the present study, we similarly found that rat primary pancreatic islet cells and βTC3 cell incubated with IHG for 72h significantly decreased the GSIS by about 55% over NG group and 15% more than SHG group. Moreover, IHG caused reduction of GSIS and insulin secretion index gradually in a time-dependent manner. The levels of insulin section were significantly decreased in primary pancreatic islet cells and βTC3 cells subjected to IHG and SHG, and IHG was lower than SHG. In CHS treatment groups, the insulin secretion index and GSIS were significantly improved and the secretion activity of islet cells was restored. These results indicated that IHG was more harmful than SHG on insulin secretion activity, and CHS could restore the secretion activity of islet cells.

β cell death which is induced by glucose toxicity is one important characteristic features of DM. Many reports had showed that chronic exposure to high glucose increases pancreatic islet beta cells death *in vitro* and *in vivo* [26, 27]. Human islets subjected to IHG for 5 days showed significantly increase in cell apoptosis [24, 28]. In this study, cell viability and apoptosis rate were measured. Our results showed the similar effects of IHG on cell viability of primary pancreatic islet cells and βTC3 cell. The cells exposed to SHG and IHG showed lower cell viability, higher apoptosis rate compared with NG group, which was accompanied by the caspase-dependent apoptosis. In living cells, lactate dehydrogenase (LDH) is present in cytoplasm and released into the extracellular when it is damaged or death [29]. So, LDH leakage is always used as a biomarker for cellular cytotoxicity. LDH leakage was induced by IHG and SHG, and inhibited by CHS treatment. The results showed that CHS protected β cells from IHG-induced apoptosis through inhibiting the expression of pro-apoptosis proteins (Bax, Bad, cleaved-caspase-9, cleaved-caspase-3 and cleaved-PARP-1).

Under normal condition, preexisting beta cells will proliferate to new derive pancreatic beta cells according to the metabolic requirements, however, this proliferation ability is attenuated in diabetes [20, 30–32]. High glucose especially IHG resulted from beta cell deficiency can conversely influence the proliferation of beta cell. In this study, islet cell proliferation was quantified by labeling primary pancreatic islet cells and βTC3 cell with 5-bromo-2-deoxyuridine (BrdU), a thymidine analog which serves as a proliferation marker. The study herein observed that IHG and SHG treatment induced reduction of BrdU positive cells, suggesting IHG would induce cell cycle arrest in βTC3 cell. The CHS treatment groups showed higher BrdU positive ratio, indicating that CHS promoted proliferation of islet cells. As reported, cell cycle is regulated by a series of cell cycle proteins, such as cyclinD1, Skp2, p21 and p27. The main function of cyclinD1 is to promote cell proliferation in G1 phrase and keep high levels until mitosis. The overexpression of cyclinD1 would induce a higher rate of β cell proliferation in vivo [33]. P21 and p27 were the main negative regulators of cell cycle, and regulated beta cell proliferation by arresting the cell cycle [34, 35]. Skp2 promoted p21 and p27 ubiquitinylation, thus induced cell proliferation. In this study, IHG and SHG treatment decreased the expression of cyclinD1 and Skp2 and increased the expression of p27 and p21, consistent with the results of cell cycle analysis. CHS could reverse this inhibitory effect of IHG and SHG through promoting the expression of cyclinD1 and Skp2 and decreasing the expression of p27 and p21.

Previous study had showed that TCF7L2 could be used as a new target for the treatment of DM [9]. TCF7L2 protects β cell from apoptosis and promotes cell proliferation, and has direct role in controlling β cell function through regulating insulin release and glucose homeostasis [11, 36]. To illuminate the protective effects of CHS on β cell apoptosis, the expression of TCF7L2 was measured. As the results showed, CHS pretreatment significantly induced the protein and gene expression of TCF7L2. The dysfunction of TCF7L2 influences the progress of DM through altering insulinotropic hormone glucagon-like peptide 1 (GLP-1) and glucose-dependent insulinotropic polypeptide (GIP) secretion or through a defective or dysfunctional GLP-1 and GIP induced insulin secretion [37]. The impaired or dysfunctional GLP-1 and GIP would reduce the level of postprandial insulin secretion, and further influence beta cell growth and beta cell differentiation [38, 39]. In this study, we found that the expression of GLP-1R and GIPR were decreased after IHG treatment, these results were consistent with TCF7L2 reduction. In DM mouse model, we also found that the levels of GLP-1 and GIP in serum and protein expression levels of GLP-1R and GIPR were reduced significantly, and CHS treatment could restore their expression levels *in vitro* and *in vivo*. Knock down the TCF7L2 by siRNA caused significantly reduction in glucose- and GLP-1 induced insulin secretion compared with CHS treatment group.

P53 is a homotetrameric transcription factor which could induce DNA repair, cell cycle arrest and apoptosis, and it is a downstream of TCF7L2 [40, 41]. In cell cycle at G1 phase, p53 enhance the transcription of p21 and then inhibit the activity of CDK, thus cause cell cycle arrest [42]. Because of the link between p53 and TCF7L2, we also confirmed whether p53 inhibition by CHS treatment was TCF7L2 dependent. When cells subjected to IHG and SHG, the expression levels of p53 were increased, and decreased in CHS treatment group. SiTCF7L2 also abolished the regulation effects on p53, cyclinD1, skp2, p21 and p27 expression by CHS treatment. These results suggested that CHS inhibited the apoptosis of β cell through increasing TCF7L2 expression and it’s downstream.

Next, we wanted to clarify how CHS induced the expression of TCF7L2. Many repots had showed the relationship between TCF7L2 and Wnt in many cells, including islet cells. The important role of Wnt signaling had been clearly clarified, and it was also involved in the regulation of metabolic homeostasis and hormone gene expression [37, 43, 44]. When the Wnt signaling pathway was blocked or overactivated might lead to tumors, T2DM or other disorders [45]. The major effector of the classical Wnt signaling pathway is the bipartite transcription factor β-catenin/TCF, which is formed by β-catenin and TCF family (TCF1-4), including TCF7L2 which also known as TCF-4 [46]. When a Wnt ligands is secreted and bings to Frizzled receptor and LRP5/6 co-receptor, the complex containing APC, Axin, GSK-3β and β-catenin is disrupted, and then β-catenin transfers from cytoplasm into nucleus, leads to the formation of β-catenin/TCF complex, and the activation of TCF or Wnt downstream target genes [47]. In this study, we found that CHS induced the gene and protein expression of Wnt3a, promoted the nuclear translocation of β-catenin which was accumulated in the cytoplasmic fraction by IHG treatment. IHG treatment also led to the decreased expression of Wnt target genes (Axin-2, c-Myc, Naked-1) and the phosphorylation levels of GSK-3β, whereas CHS induced the expression of Axin-2, c-Myc, Naked-1 and the phosphorylation GSK-3β. These results indicated that CHS had activation effects on the Wnt/β-catenin pathway. To further study the relationship between Wnt/β-catenin and TCF7L2, a Wnt/β-catenin antagonist XAV-939 was used. We found that XAV-939 abolished the effects of CHS on TCF7L2, GIPR, cyclinD1 and skp2 expressions, also decreased cell viability, BrdU positive ratio and glucose induced insulin release levels. Taken together, the results showed that CHS regulated the Wnt/GSK-3β/β-catenin axis and thereby the expression of TCF7L2 target genes.

HMG-box containing protein 1 (HBP1), a member of the high mobility group (HMG) protein family, plays important roles in regulating cell cycle, apoptosis and terminal differentiation in many tissues and cells [48, 49]. In cell nucleus, HBP1 competes with TCF to bind with β-catenin for transcription of target genes and inhibits the activity of β-catenin/TCF which induces the inhibition of Wnt/β-catenin pathway [50]. Previous work had shown that HBP1 repressed the expression of cyclin D1 and c-Myc genes to inhibit G1 progression in cells [51]. Even through the relationship between HBP1 and Wnt had been studied in other tissue, especially tumor tissues, its role in islet cells were largely unknown to us. In this study, we found that HBP1 expression level was enhanced in βTC3 cells by SHG or IHG treatment and in T2DM mouse model, together with the inhibition of Wnt/β-catenin pathway. CHS treatment inhibited the expression of HBP1 *in vitro* and *in vivo*. Further studies showed that knockdown of HBP1 had a synergistic effect with CHS and overexpression of HBP1 abolished the effects of CHS. These results suggested that the regulation effects of CHS on Wnt/β-catenin/ TCF7L2 might through inhibiting HBP1 expression.

In conclusion, we proposed a novel mechanism for the effects of CHS on β cell apoptosis and proliferation. In this model, CHS treatment induced the secretion of GLP-1 and GIP, inhibited the cell apoptosis and promoted cell proliferation, thus protected islet cells from IHG and SHG induced injuries *in vivo* and *in vitro*. HBP1/Wnt/β-catenin/TCF7L2 was the core component for the functions of CHS. CHS inhibited HBP1 expression and activated Wnt/β-catenin/TCF7L2 pathway which induced the secretion of GLP-1 and GIP, inhibited the caspase related apoptosis, and promoted cell proliferation (Figure 8I). Our data supported that HBP1/Wnt/β-catenin/TCF7L2 could be used as a target for DM treatment. In previous studies, we had studied the safety of CHS in treating DM, and in this study, we performed the possible mechanism of CHS during preventing β cell from SHG inducing injuries *in vivo* and *in vitro*. These results demonstrated that CHS might be clinically applied for DM.

## MATERIALS AND METHODS

### Drugs and reagents

Chikusetsu saponin IVa (CHS) was isolated from *Aralia taibaiensis* and obtained from the New Drug Research and Development Center, Fourth Military Medical University. Cell counting kit 8 (CCK8) was obtained from 7sea Biotechnology (Shanghai, China). Dulbecco’s modified Eagle’s medium (DMEM) and trypsin were purchased from GIBCO (Life Technologies, Grand Island, NY). Fetal bovine serum (FBS) was purchased from Sijiqing Biotechnology (Zhejiang, China). Fluorescein annexin V-FITC/PI double labeling kit and cell cycle detection kit were purchased from Nanjing Jiancheng Bioengineering Institute (Nanjing, People’s Republic of China). The Bio-Rad imaging system for western blots was purchased from Bio-Rad (Hercules, CA, USA). Bax (No. 2772), Bcl-2 (No. 3498), Bad (No. 9292), Cleaved-caspase 3 (No. 9661), Cleaved-PARP (No. 94885), Cytochrome c (No. 11940), β-actin (No. 4970), VDAC (No. 12454), Cyclin D1 (No. 2978), Skp2 (No. 4313), p21 (No. 2947), p27 (No. 3688), p53 (No. 2524), Wnt3a (No. 2721), β-Catenin (No. 8480), c-Myc (No. 9402) antibodies were purchased from Cell Signaling Technology (Beverly, MA, USA). Cleaved-caspase 9 (sc-133109), TCF7L2 (sc-271287), GLP-1R (sc-390774), HBP1 (sc-515281) antibodies were purchased from Santa Cruz (CA, USA). GIPR (AB-2720164), Axin-2 (PA5-78247), Naked-1(PA5-17389) antibodies were purchased from Invitrogen (Carlsbad, CA). The goat anti-rabbit and goat anti-mouse secondary antibodies were purchased from Boster Biological Technology co.Itd (Wuhan, China). The bicinchoninic acid (BCA) protein assay kit was obtained from Beyotime Biotechnology (Shanghai, China). The siRNAs and LipoFiter liposomal transfection reagent were purchased from Hanbio Biotechnology (Shanghai, China). TCF7L2 siRNA was purchased from Santa Cruz Biotechnology (CA, USA). All other chemicals and reagents used in this experiment could be obtained from commercial corporation.

### Animals and diabetic models

The experimental protocol was approved by the Ethics Committee for Animal Experimentation and was performed according to the Guidelines for Animal Experimentation of the Fourth Military Medical University and the National Institute of Health Guide for the Care and Use of Laboratory Animals (NIH Publications No. 80-23) revised in 2011. The mice and β-catenin gene knockout mice (β-catenin^-/-^) were provided by the Experimental Animal Center of the Fourth Military Medical University. Animals were housed in a temperature and humidity-controlled room and freedom to food and water during experiments.

After one week of adaptive feeding, four-week male mice (14.5±0.46g) were randomly divided into diabetes group and normal control group. The mice in diabetes group were fed with high-fat diet (HFD) for 8 weeks, and given with streptozotocin (STZ, Sigma, St Louis, MO, USA) at a dose of 50 mg/kg dissolved in 100 mM citrate buffer pH 4.5 through intraperitoneal injection. After 4 weeks, fasting blood glucose levels were measured using Bayer's BREEZE2 meter by caudal veins blood. Mice with blood glucose levels of >11.1 mM were regarded to be successful model of diabetes. The blood glucose levels in diabetes group and normal control group were measured at the same time points using same processes and the fasting blood glucose coefficient of variation (FBG-CV= interday blood glucose means/standard deviation) was calculated as reported [1]. Mice with higher FBG-CV values (two fold more than that in control group) were considered fluctuant high blood glucose in DM mice. The DM mice were randomly allocated into model group, CHS treatment group and positive control rosiglitazone (Ros) treatment group. CHS (120 mg/kg) or Ros (2 mg/kg) intervention was initiated after 12 weeks. The control group was given vehicle. Drugs or vehicle was given for additional 30 days. A total of 10 mice in each group were used. After experiments, the blood samples and islet tissues were collected and stored at -80°C until further analysis.

### Cell culture

The βTC3 cell line was obtained from China Center for Type Culture Collection (Shanghai, China) and cultured in RPMI 1640 medium with 11 mmol/L D-glucose supplemented with 10% fetal bovine serum (FBS), 100 μg/mL streptomycin, 100 U/mL penicillin, 1 mmol/L sodium pyruvate, 2 mmol/L L-glutamine, 10 mmol/L HEPES, and 50 μmol/L 2-mercaptoethanol at 37°C in a humidified incubator with 5% CO_2_. The culture medium was replaced every other day.

Primary pancreatic islet cells were isolated from male SD rats according to the method of Gotoh et al [52]. The pancreas were cut into pieces and digested gradually by collagenase P, and then separated on a discontinuous Ficoll gradient. The primary pancreatic islet cells were cultured in RPMI 1640 medium with 11 mmol/L D-glucose supplemented with 10% FBS, 1 mmol/L sodium pyruvate, 10 mmol/L HEPES, and 50 μmol/L 2-mercaptoethanol at 37°C in a humidified incubator with 5% CO_2_.

Primary pancreatic islet cells and βTC3 cells were grown until a confluency of 90%, washed by phosphate buffer, and dispersed with trypsin/EDTA when subcultured in appropriate flasks or plates according to the experiment performed. Cells were divided into six groups: (1) control group: cells were cultured in the normal concentration of glucose (NG, 11.1 mmol/L); (2) sustained high glucose group (SHG): cells were cultured in the sustained high glucose (33.3 mmol/L); (3)intermittent high glucose group (IHG): cells were cultured in the 11.1 and 33.3 mmol/L glucose alternating every 12 h; (4) NG+CHS: cells were pretreated with CHS (40μM) for 24h before NG treatment; (5) SHG+CHS: cells were pretreated with CHS (40μM) for 24h before SHG treatment; (6) IHG+CHS: cells were pretreated with CHS (40μM) for 24h before IHG treatment. Cells were maintained in the required conditions for 72 h, and the culture media were changed every day. All experiments were repeated for three times.

### Cell viability

Cell viability was measured by the Cell Counting Kit-8 (CCK-8) according to the instruction. The cells at a density of 2 × 10^5^ cells/ml were seeded on a 96-well plate in 100μl culture medium. After cultured for 24h, the cells were treated with NG, SHG, IHG, NG+CHS, SHG+CHS, IHG+CHS for indicated time. 10μl of CCK-8 solution was added to each well before incubation for another 4h. Absorbance was determined at an absorbance of 450 nm using a microplate reader. Cell viability was expressed as a percentage of cytoprotection, versus the NG group set at 100%.

### LDH cytotoxicity assay

Once the cells and cell membranes are damaged, lactate dehydrogenase (LDH) is released into the extracellular space, so it always be used as a biomarker for cellular cytotoxicity. In this study, LDH levels in the culture medium were measured by a LDH detection kit as the instruction direct. The cells were seeded in 6-well plate at 5 × 10^5^ cells/well for 24h and then treated with NG, SHG, IHG, NG+CHS, SHG+CHS, IHG+CHS. At each time point, the culture medium was collected and measured by LDH detection kit at 490 nm using a microplate reader. LDH leakage was calculated.

### Glucose stimulated insulin secretion (GSIS) and insulin content assay

After different treatments, primary pancreatic islet cells and βTC3 cells were washed by phosphate buffer for three times, and then pre-incubated in Krebs–Ringer bicarbonate HEPES buffer (KRBH: 115 mmol/L NaCl, 5 mmol/L KCl, 1 mmol/L MgCl_2_, 25 mmol/L HEPES, pH 7.4) supplemented with 0.1% (w/v) BSA for 1 h in order to deplete glucose. After these, fresh KRBH buffer supplemented with 3 mmol/l or 27.8 mmol/l glucose were used to incubate the cells for another 30min at 37°C. The culture medium was collected to measure the insulin level by an insulin RIA kit (Beijing Institute of Atomic Energy, China).

Total insulin content in the cells was extracted using the acid/ethanol method. Primary pancreatic islet cells and βTC3 cells were incubated in 1% hydrochloric acid alcohol for 8h at 4°C. The insulin level was measured by the insulin RIA kit.

### Detection of apoptosis

The cell apoptosis was detected by the Annexin V FITC/propidium iodide (PI) Apoptosis Kit. The βTC3 cells were seeded at 2× 10^5^ cells/well into 6-well plates and cultured at 37°C for 24h. And then, cells were treated by NG, SHG, IHG, NG+CHS, SHG+CHS and IHG+CHS for 72h, washed in phosphate buffer and collected. Cells were resuspended in 400μl binding buffer, and FITC-conjugated annexin-V and PI were added and incubated in the dark for 10min. Finally, another 100ul was added and the apoptotic rates were recorded by flow cytometric (BD, USA).

### Detection of cell proliferation

The βTC3 cell proliferation was measured by a 5-Bromo-2’-deoxy-uridine (BrdU) labeling and detection kit (Roche, Indianapolis). Briefly, cells were plated onto cover-slide and incubated at the appreciated condition. After addition of BrdU into the medium, cells were cultured for 1 h and then fixed and permeabilized, incubated with an anti-BrdU antibody (1:100) at 37°C for 120min. HRP-conjugated goat anti-rat IgG was used to incubate the cells for 60min at 37°C followed by DAB and hematoxylin staining. The samples were then observed by an invert microscope. 10 different horizons were counted and the Brdu positive cell ratio was calculated.

### Morphological analysis of nuclear

To further analysis the nuclear morphology, a nuclear dye DAPI was used to stain the cells. Cells were plated onto cover-slide and incubated at the appreciated condition, then stained with DAPI (5 μ g/mL) for 30 min at 37°C in the dark. Phosphate buffer was used to wash the cells for three times, and then the immunofluorescent assay was measured by an Olympus C2 confocal microscopy.

### Small interfering RNA transfection

Specific short interfering RNA (siRNA) molecules targeted with HBP1 and TCF7L2 were chemically synthesized by Shanghai Genechem Company. The control group was given a nonspecific scrambled siRNA. The cells were transfected with scrambled siRNA or HBP1 and TCF7L2 -targeted siRNA (30 nM) using the Lipofectamine 2000, according to the manufacturer’s protocol. Following 48-h transfection, βTC3 cell were pretreated with CHS and then subjected to different treatments.

### Quantitative real-time PCR analysis

After different treatments for 72 h, total cellular RNA of primary pancreatic islet cells and βTC3 cells were extracted using Trizol reagent (Invitrogen, Carlsbad, CA). For RT-PCR, 2.0μg of total RNA was then reversely transcribed into cDNA with RT-PCR kits. The QuantiTect SYBR green PCR kit (Qigan, Inc. Valencia, CA) was used to quantitate reverse transcription-PCR on an iCycler iQ real-time PCR detection system (Bio-Rad). The PCR was performed for 40 cycles at 95°C for 15s, 60°C for 30s, 72°C for 30s. All transcript levels were corrected with that of the housekeeping gene β-actin. The results were presented as fold over the control.

### Western blotting analysis

After different treatments, cells were washed by cold PBS and lysed at 4°C with RIPA buffer supplemented PSMF (1mmol/L). The protein concentration in each samples were measured by a BSA kit. Equal amounts of nuclear or total protein (25μg) were separated on 10% sodium dodecyl sulfate-polyacrylamide gel electrophoresis (SDS-PAGE) and transferred on PVDF membranes. After the membranes were washed by TBS and blocked with 5% nonfat milk for 1h, and then incubated in the presence of different primary antibodies (anti-Wnt-3a, anti-β-catenin, anti-TCF7L2, anti-Bcl2, anti-Bax, anti-cleaved caspase 3, anti-Bad, anti-c-Myc, anti-cyclin D1, anti-skp2, anti-HBP1, anti-p53, anti-p27, anti-p21, anti-GLP-1R, anti-GIPR, anti-P-GSK-3β, anti-cytochrome c, anti-PARP, anti-cleaved caspase 9, anti-Axin, anti-Naked-1 and anti-β-actin) at 4°C overnight. The membranes were incubated with horseradish peroxidase-conjugated secondary antibody for 1 h at room temperature. The bands were washed with TBST and developed by ECL chemiluminescence method. Optical densities of the bands were scanned with a Bio-Rad imaging system (Bio-Rad, Hercules, CA, USA) and quantified using the Image Lab software package (Bio-Rad, Hercules, CA, USA). β-actin and lamin B served as internal control in cytoplasm and nuclear respectively.

### Statistics

All Data were expressed as the means ± standard error of mean (SEM) from at least three repeated experiments. Statistical significance (*P*<0.05) for each variable was evaluated by one-way ANOVA followed by the Bonferroni correction by GraphPad Prism 5.0. All of the groups were analyzed simultaneously using the LSD t-test.
